# Collateral Damage: How Medicaid Cuts Under the One Big Beautiful Bill Act Threaten Rural Radiation Oncology

**DOI:** 10.7759/cureus.103553

**Published:** 2026-02-13

**Authors:** Drishti Panse, Monica Wassel, Catherine Yu, Jared P Rowley, Kunal K Sindhu

**Affiliations:** 1 Radiation Oncology, Icahn School of Medicine at Mount Sinai, New York City, USA; 2 Radiation Oncology, Maimonides Medical Center, Brooklyn, USA

**Keywords:** healthcare access, hospital closures, medicaid policy, radiation oncology, rural health

## Abstract

Rural communities face longstanding gaps in access to cancer care, and many hospitals continue to operate with limited oncology staffing and services. With the One Big Beautiful Bill Act introducing major Medicaid cuts, these fragile systems may come under greater pressure. This editorial examines how reduced coverage could further destabilize rural hospitals, widen delays in cancer diagnosis and treatment, and intensify workforce challenges, while also outlining policy approaches that might help preserve access for patients who already face the steepest barriers.

## Editorial

Cancer is the second leading cause of death in the United States, and radiation therapy (RT) plays a critical role in the treatment of many malignancies. Unfortunately, access to RT is unevenly distributed. Sociodemographic and geographic disparities have been linked to RT nonadherence and poorer survival outcomes among rural patients with cancer. Medicaid is a key source of health care coverage for low-income populations, including many rural residents. Previous studies have demonstrated that Medicaid expansion under the Affordable Care Act improved rates of timely cancer screening and diagnosis, increased access to cancer-directed treatments, and contributed to improved survival rates [1].

The One Big Beautiful Bill Act (OBBBA), signed into law by President Trump on July 4, 2025, permanently extends the individual tax rates in the Tax Cuts and Jobs Act of 2017, increases border security and military funding, and rolls back clean energy subsidies that were enacted via the Inflation Reduction Act of 2022, among other provisions. To partially offset these expenditures, the bill outlines a series of spending cuts that include substantial reductions in federal Medicaid spending. As a consequence, the Congressional Budget Office (CBO) has estimated that the bill would result in 10 million fewer Americans with Medicaid coverage by 2034 [2].

By slashing Medicaid funding, the OBBBA has the potential to disproportionately affect rural hospitals. These pressures are occurring at a time of uncertainty for fragile rural health systems, as the Centers for Medicare and Medicaid Services pursues payment reforms, such as the Transforming Episode Accountability Model (TEAM), which introduces prospective bundled payments for selected hospital-based episodes. Rural hospitals often operate with fewer health care resources and higher fixed costs, face persistent challenges in recruiting and retaining clinicians, and are more likely to serve uninsured patients or patients who are covered by public insurance programs. As a result, they are at a higher risk of closure as compared to their urban counterparts. Unfortunately, each such closure can result in devastating consequences by forcing patients to travel longer distances to obtain care, which has been associated with lower rates of cancer screening and delayed treatment. In the absence of a legislative fix, the OBBBA risks widening existing disparities in access to oncologic care, particularly for rural and underserved patients.

To better understand the potential impact of the OBBBA, we evaluated the availability of medical and radiation oncology services in rural hospitals most at risk from the bill’s Medicaid cuts. We adopted criteria outlined in the “Letter on Rural Hospitals,” authored by Senators Edward Markey (D-Massachusetts), Ron Wyden (D-Oregon), Jeff Merkley (D-Oregon), and Charles Schumer (D-New York), which identified 338 hospitals that either are in the top 10% of Medicaid payer mix among rural hospitals nationally or have experienced three consecutive years of negative total margins [3]. We assessed whether these hospitals had on-site radiation or medical oncology services or access to infusion sites within a 10-mile radius of the hospital. We additionally quantified the number of practicing radiation and medical oncologists at each facility.

We identified 41 radiation oncologists, representing approximately 9% of rural radiation oncologists nationwide, 131 medical oncologists, and three and 35 radiation and medical oncology advanced practice providers, respectively, employed at these at-risk hospitals. Twenty-seven hospitals (8%) offered infusion services without an associated oncology provider, 27 (8%) employed at least one radiation oncologist, and 71 (21%) employed at least one medical oncologist. The median number of radiation and medical oncologists employed per hospital was one, with a range of one to five for radiation oncologists and one to seven for medical oncologists. Among these hospitals, 18 employed one radiation oncologist and 36 employed one medical oncologist. These findings are consistent with prior studies showing lower oncologic density outside of metropolitan areas and a limited healthcare workforce in rural areas.

**Figure 1:**
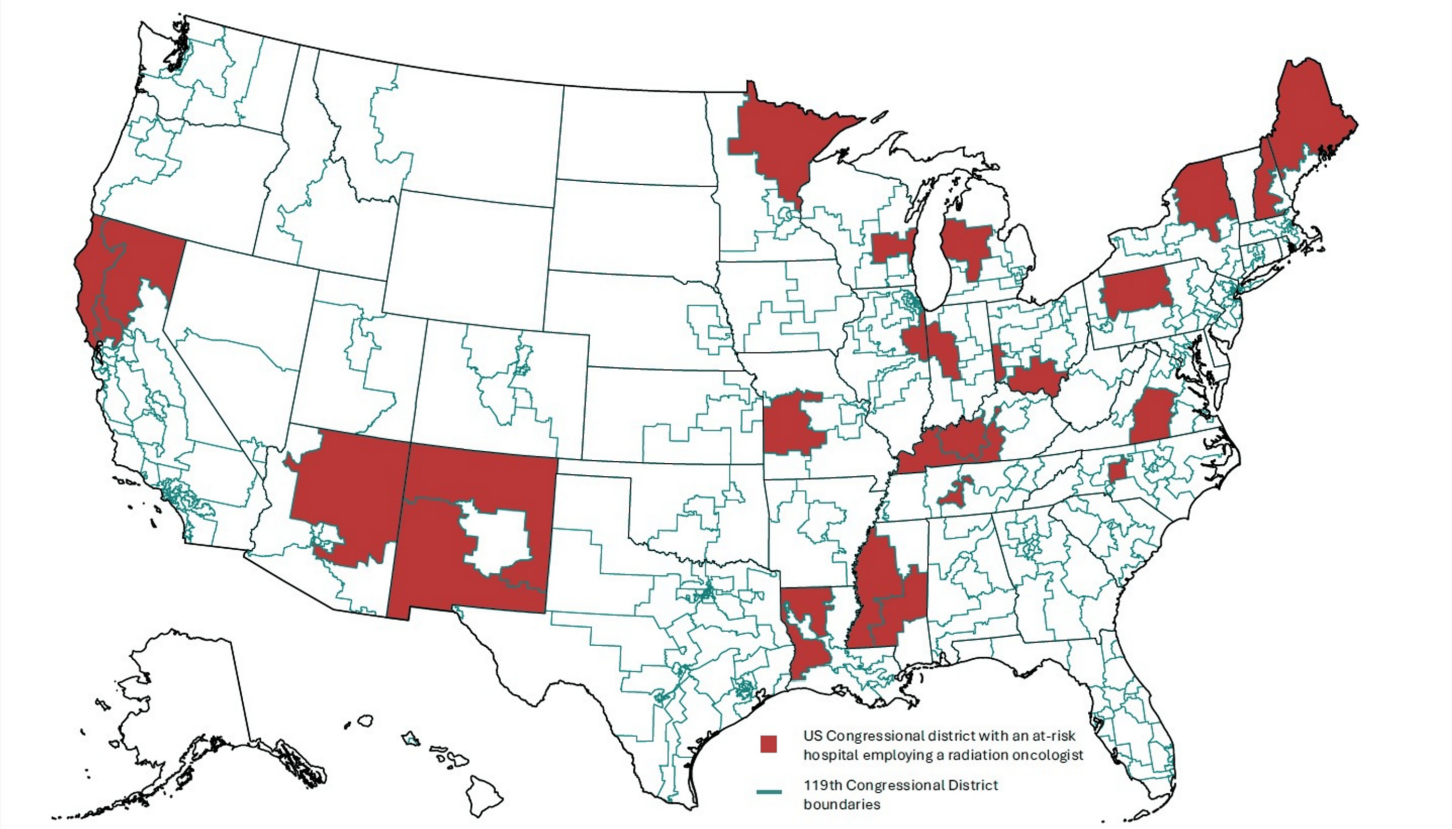
U.S. congressional districts with an at-risk hospital employing a radiation oncologist Image credit: Authors

These hospitals were located in 37 (74%) states, 23 of which voted for the Republican candidate and 14 for the Democratic candidate in the 2024 presidential election. They were also distributed across 68 (16%) congressional districts, 58 of which voted for the Republican candidate and 10 for the Democratic candidate in the 2024 presidential election. Twenty-six and 49 of these congressional districts had an at-risk hospital that employed at least one radiation and medical oncologist, respectively. Figure 1 shows a map, generated using Observable Plot, of the 26 U.S. congressional districts with an at-risk hospital employing a radiation oncologist (119th congressional district delineations were downloaded from the U.S. Census Bureau).

Collectively, per 2024 estimates from the U.S. Census Bureau, the districts that house at least one medical or radiation oncology provider that we identified are home to 79,724,092 people, representing 23.4% of the population of the United States. The 17 rural congressional districts containing at-risk hospitals with just one employed radiation oncologist reported a combined population of 13,625,439 residents, underscoring the large number of individuals who may be affected by the loss of Medicaid coverage and decreased access to oncology services.

Taken together, our findings suggest that federal cuts to Medicaid will disproportionately impact rural hospitals already struggling to sustain oncology services. By reducing operating margins, these cuts are likely to accelerate hospital closures and thus lead to decreased access to cancer care. Americans living in rural communities across the country will be at risk of delays in cancer diagnosis, referral, and treatment due to a loss of oncology and primary care providers, diagnostic imaging services, and infusion centers. In doing so, these changes have the potential to delay the initiation of RT courses, which has been associated with decreased survival [4].

Patients displaced from financially at-risk hospitals are also likely to seek care at neighboring facilities, further straining the healthcare ecosystem in these regions. These regions are already susceptible to workforce shortages, as rural communities attract a smaller fraction of practicing radiation and medical oncologists [5]. This may lead to further delays in care and diagnosis for all patients at the remaining centers and contribute to financial toxicity for both patients and institutions alike. Most critically, our findings represent only a floor for the potential impact of the bill. Hundreds of rural hospitals, while not necessarily qualifying for inclusion in the letter issued by the senators, operate with low margins and will be squeezed in the coming years as implementation of the OBBBA proceeds. Oncology services are unlikely to be spared from cuts. While emerging care-delivery models, including expanded roles for non-physician clinicians, may ultimately improve efficiency in some settings, their adoption remains uneven and often depends on local regulatory, training, and reimbursement environments.

At a time when rural communities already face higher rates of cancer-related mortality and worse treatment outcomes, creative solutions will be needed to counter the expected impact of the OBBBA’s Medicaid cuts. While insufficient on its own and not a substitute for comprehensive value-based payment reform, the Radiation Oncology Case Rate (ROCR) Act may help to partially address these new challenges by offering greater payment predictability for RT services in resource-constrained settings. The ROCR Act proposes a shift from per-radiation treatment reimbursement to an evidence-based, case rate-based payment model. This approach allows Medicare reimbursement to be more patient-centered, supporting shorter RT courses and helping to reduce cost and transportation barriers. Additionally, expanding telehealth and tele-oncology resources, implementing educational initiatives to support rural providers, incentivizing workforce redistribution, and increasing access to clinical trials may help soften the blow. However, policymakers must act quickly. In the absence of a legislative fix, the OBBBA is likely to further exacerbate the already significant strains on rural hospitals, the closures of which will only worsen the burdens faced by rural patients with cancer in accessing care.
